# Effect of a rescue or recurrence dose of lasmiditan on efficacy and safety in the acute treatment of migraine: findings from the phase 3 trials (SAMURAI and SPARTAN)

**DOI:** 10.1186/s12883-019-1420-5

**Published:** 2019-08-13

**Authors:** Li Shen Loo, Brian M. Plato, Ira M. Turner, Michael G. Case, Joel Raskin, Sherie A. Dowsett, John H. Krege

**Affiliations:** 1 Eli Lilly and Company, Corporate Center, Indianapolis, IN 46285 USA; 2 Norton Neuroscience Institute, 3991 Dutchmans Ln #310, Louisville, KY 40207 USA; 3 The Center for Headache Care and Research, Island Neurological Associates PC, An affiliate of ProHealthcare, 824 Old Country Rd, Plainview, NY 11803 USA

**Keywords:** Lasmiditan, Rescue, Recurrence, Second dose, Phase 3

## Abstract

**Background:**

We studied the efficacy and safety of a second dose of lasmiditan for acute treatment of migraine.

**Methods:**

SAMURAI and SPARTAN were double-blind, placebo-controlled Phase 3 studies in which individuals with migraine were randomized to oral lasmiditan 50 mg (SPARTAN only), 100 mg, 200 mg, or placebo. Study drug was to be taken within 4 h (h) of onset of a migraine attack (moderate or severe pain). A second dose of study drug was provided for rescue (patient not pain-free at 2 h and took a second dose 2-24 h post-first dose) or recurrence (patient pain-free at 2 h, but experienced recurrence of mild, moderate, or severe migraine pain and took a second dose 2-24 h after first dose). Randomization to second dose occurred at baseline; patients originally assigned lasmiditan were randomized to the same lasmiditan dose or placebo (2:1 ratio), and those originally assigned placebo received placebo. Data from SAMURAI and SPARTAN were pooled for efficacy and safety assessment of a second dose of lasmiditan.

**Results:**

The proportion of patients taking a second dose was lower with lasmiditan versus placebo, and decreased with increasing lasmiditan dose; the majority who took a second dose did so for rescue. In patients taking lasmiditan as first dose, outcomes (pain free, most bothersome symptom [MBS] free) at 2 h after a second dose for *rescue* were similar whether the second dose was lasmiditan or placebo (*p* > 0.05 in all cases). In patients taking lasmiditan for first dose, outcomes at 2 h after a second dose for *recurrence* were as follows: lasmiditan pooled versus placebo - pain free, 50% vs 32% (*p* > 0.05); MBS free, 71% vs 41% (*p* = 0.02); pain relief, 77% vs 52% (*p* = 0.03). In patients whose first dose was lasmiditan, the incidence of treatment emergent adverse events (TEAEs) reported after the second dose was similar whether second dose was lasmiditan or placebo.

**Conclusions:**

A second dose of lasmiditan showed some evidence of efficacy when taken for headache recurrence. There was no clear benefit of a second dose of lasmiditan for rescue treatment. The incidences of TEAEs were similar whether the second dose was lasmiditan or placebo.

**Trial registration:**

SAMURAI (NCT02439320) [April 2015]. SPARTAN (NCT02605174) [May 2016].

## Background

Acute therapy for migraine is designed to relieve pain and disability, stop attack progression, and restore functioning [1]. The ultimate goal of acute treatment is consistent, rapid, and complete relief without headache recurrence, with little or no use of backup or rescue medications [2]. Unfortunately, the response to acute treatment is inadequate in some patients, and for those who experience an initial response, there is symptom recurrence [3, 4].

The need for a second dose of therapy, or alternate therapy, for persistence of symptoms or their recurrence (therapy for rescue or recurrence, respectively) is key to determining the overall pharmacoeconomic benefit of therapy; if a second dose is required, it is important to understand its benefit, beyond the first dose, as well as any additional risks incurred.

Lasmiditan is a centrally-penetrant, highly selective, oral 5-HT_1F_ receptor agonist currently under development as an acute therapy for migraine [5]. Two phase 3 single migraine attack studies of lasmiditan, SAMURAI and SPARTAN, have been completed [6, 7]. In these studies, treatment with lasmiditan (all doses) resulted in a significant increase in the proportion of patients who were headache pain-free (primary endpoint) or most bothersome symptom (MBS)-free (key secondary endpoint) at 2 h (*p* < 0.01 versus placebo in all cases). The percentages headache pain-free at 2 h for the lasmiditan 200 mg group versus placebo were 32% versus 15% in SAMURAI and 39% versus 21% in SPARTAN.

SAMURAI and SPARTAN were almost identical in design. In both studies, a second dose of study drug was permitted for rescue or recurrence. The purpose of the current study is to describe the second dose population and the efficacy and safety findings for a second dose of lasmiditan, using data from these 2 phase 3 studies.

## Methods

### Study designs

SAMURAI and SPARTAN [6, 7] were randomized, double-blind, placebo-controlled phase 3 trials designed to study the effects of lasmiditan in the acute treatment of migraine; both studies were conducted in the United States, and SPARTAN also included study sites in the United Kingdom and Germany. The studies were almost identical in design. Key inclusion criteria were as follows: Migraine Disability Assessment score of at least 11 indicating moderate or severe migraine-associated disability; history of migraine of at least 1 year; 3–8 migraine attacks per month with less than 15 headache days per month; and migraine onset before 50 years of age.

For first dose of study drug, patients were randomized equally to lasmiditan 50 mg (SPARTAN only), 100 mg, 200 mg, or placebo, to be taken on an outpatient basis. Patients were instructed to take study drug within 4 h of onset of a migraine attack with a headache of at least moderate severity and not improving. A second dose was taken 2 to 24 h after first dose if needed for rescue or recurrence. Patients taking lasmiditan as a first dose were randomized in a 2:1 ratio to the same dose of lasmiditan or placebo, and patients taking placebo as a first dose received a second dose of placebo; randomization to the second dose occurred at the same time as randomization to first dose. The rescue population referred to patients who did not achieve headache pain-free status at 2 h, completed the 2-h assessments, and took a second dose of study drug between 2 and 24 h post-first dose. The recurrence population referred to patients who achieved headache pain-free status at 2 h but then experienced recurrence of mild, moderate, or severe migraine pain and took a second dose of study drug up to 24 h from the first dose.

The primary and key secondary endpoints in SAMURAI and SPARTAN were the proportion of patients who were headache pain-free and the proportion who were MBS free at 2 h post-first dose.

This paper adheres to CONSORT guidelines.

### Outcome measures

Patients were asked to record in the electronic diary their headache pain severity as none, mild, moderate, or severe and whether they were experiencing nausea, phonophobia, photophobia, or vomiting at baseline, at half hour intervals up to 2 h and at 3, 4, 24, and 48 h after dosing. Patients identified at baseline their most bothersome migraine associated symptom out of nausea, phonophobia and photophobia. Patients were also asked the following questions: 1) “*How much is your migraine interfering with your usual activities?*” (asked at all time points, responses reported using a 4-point numeric rating scale); and 2) the Patient Global Impression of Change (PGIC) question [8], “*How do you feel after taking study medication*” (asked at 2 h post dose, responses recorded using a seven-point Likert scale from “very much better” to “very much worse”).

### Adverse event collection

At 0.5, 1, 1.5, 2, 3, 4, 24, and 48 h after the first dose and again at the same intervals after a second dose of study drug, if taken, an electronic diary was employed to ask “*Do you feel anything unusual since taking the study medication that you have not felt with a migraine before?*” If the patient answered “yes”, they were to receive a call from the site to determine if an adverse event (AE) had been experienced. Those AEs that occurred or worsened within 48 h of dosing were considered treatment-emergent.

### Statistical analysis

Efficacy hypotheses were that, after a first dose of lasmiditan, a second dose of lasmiditan would be superior to a second dose of placebo when taken for rescue or when taken for recurrence. Since the number of patients choosing to take a second dose of study medication could not be determined prior to the conduct of the study, no formal power analyses were conducted.

Efficacy analyses were performed in the intention-to-treat (ITT) second dose population, which was defined as patients who used a second dose of study drug to treat a migraine and had any post-second dose headache severity or symptom assessments. This population was further classified as ITT second dose rescue population (not pain-free at 2 h and took a second dose of study medication) and ITT second dose recurrence population (pain-free at 2 h and took a second dose of study medication). Safety analyses utilized the second dose safety population, which was defined as all patients who used 2 doses of study drug, regardless of whether they completed any study assessments.

Mantel-Haenszel odds ratios (ORs) for efficacy outcomes were computed and compared between lasmiditan and placebo, with study as a covariate. For treatment comparisons, an estimate of the OR of achieving a response, as well as the corresponding confidence interval (CI) and *p*-value using Wald’s test, was computed. Time to second dose graphics were created using Kaplan-Meier analyses. Continuous variables were summarized using descriptive statistics; categorical variables were summarized using counts and percentages. All efficacy endpoints were tested at a 2-sided significance level of 0.05.

To enable some level of comparison of pain recurrence findings for lasmiditan with those for triptans, we performed a post hoc sensitivity analysis using data from SAMURAI and SPARTAN and an alternate definition of recurrence to be consistent with prior studies [4].

Analyses were performed using SAS software, version 9.4/EG 7.1 (SAS Institute, Cary, NC).

## Results

### Study populations and patient characteristics

The proportion of patients taking a second dose was lower with lasmiditan versus placebo, and the proportion decreased with increasing lasmiditan dose (placebo, 60%; lasmiditan 50 mg, 46%; lasmiditan 100 mg, 43%; lasmiditan 200 mg, 36%). Second dose populations are detailed in Table 1. The vast majority taking a second dose did so for rescue.

**Table 1:** Second dose study populations

FIRST DOSE →	Placebo (*N* = 1262)	Lasmiditan 50 mg (*N* = 654)	Lasmiditan 100 mg (*N* = 1265)	Lasmiditan 200 mg (*N* = 1258)
Intention-to-treat population,^a^ n	1130	598	1133	1120
Pain at 2 h, n (%)^b,c^	924 (82%)	429 (72%)	796 (70%)	724 (65%)
Took rescue dose, n (%)^d^	606 (66%)	245 (57%)	388 (49%)	310 (43%)
Pain free at 2 h, n (%)^b^	206 (18%)	169 (28%)	337 (30%)	396 (35%)
Took recurrence dose, n (%)^e^	21 (10%)	13 (8%)	35 (10%)	28 (7%)

^a^Took first dose of study drug and had any post-dose headache severity or symptom assessments

^b^Denominator = first dose ITT population

^c^Also included patients with missing data, who were assumed not to be pain free at 2 h

^d^Denominator = pain at 2 h population

^e^Denominator = pain free at 2 h population

Baseline characteristics of the study populations are shown in Table 2. Baseline characteristics in those taking 1 versus 2 doses or in second-dose rescue versus recurrence populations were generally similar.

**Table 2:** Baseline characteristics of patients taking one or two doses, and overall

	One dose Population (*N* = 2139)	Two Dose Population^a^		Overall Population^b^ (*N* = 3981)
		Rescue (*N* = 1549)	Recurrence (*N* = 97)	
Age, mean (SD), years	41.1 (12.8)	43.2 (11.8)	43.5 (12.7)	42.2 (12.4)
Female	84%	84%	88%	84%
BMI, mean (SD)	30.4 (7.9)	30.1 (9.5)	31.3 (7.1)	30.2 (8.6)
Migraine history duration, mean (SD), years	17.3 (12.6)	20.4 (12.9)	19.6 (13.3)	18.7 (12.9)
Migraine attacks/month in past 3 months, mean (SD)	5.1 (1.7)	5.4 (2.2)	5.3 (1.7)	5.2 (1.9)
Patients with history of migraines with aura	37%	41%	41%	39%
Patients using migraine reducing medications at randomization^c^	12%	20%	18%	15%

*Abbreviation*: *BMI* body mass index, *SD* standard deviation

^a^Second dose intention-to-treat population

^b^Intention-to-treat population

^c^Based on the question “*Is the subject currently using medications to reduce the frequency of migraine episodes?”* asked during randomization, but adjusted with information from concomitant medications data

There were no differences in overall pain severity at baseline between the dose groups. The baseline pain severity at first dose was similar across the dose groups in both the rescue and recurrence populations, as was the baseline pain severity at second dose. More patients dosed when pain was mild at second dose than at first dose (Table 3).

**Table 3:** Baseline pain severity at first and at second dose for rescue and recurrence populations and overall

POPULATION - Baseline pain severity	Placebo		Lasmiditan 50 mg		Lasmiditan 100 mg		Lasmiditan 200 mg	
	At First Dose	At Second Dose	At First Dose	At Second Dose	At First Dose	At Second Dose	At First Dose	At Second Dose
RESCUE	*N* = 606		*N* = 245		*N* = 388		*N* = 310	
- Severe^a^	174 (28.7)	144 (23.8)	72 (29.4)	57 (23.3)	124 (32.0)	84 (21.6)	101 (32.6)	75 (24.2)
- Moderate	428 (70.6)	332 (54.8)	170 (69.4)	127 (51.8)	258 (66.5)	179 (46.1)	206 (66.5)	152 (49.0)
- Mild	4 (0.7)	130 (21.5)	3 (1.2)	61 (24.9)	6 (1.5)	124 (32.0)	3 (1.0)	83 (26.8)
- None	–	0	–	0	–	1 (0.3)	–	0
RECURRENCE	*N* = 21		*N* = 13		*N* = 35		*N* = 28	
- Severe	4 (19.0)	1 (4.8)	3 (23.1)	3 (23.1)	6 (17.1)	5 (14.3)	10 (35.7)	7 (25.0)
- Moderate	16 (76.2)	14 (66.7)	9 (69.2)	9 (69.2)	29 (82.9)	23 (65.7)	18 (64.3)	19 (67.9)
- Mild	1 (4.8)	5 (23.8)	1 (7.7)	1 (7.7)	0	4 (11.4)	0	2 (7.1)
- None	–	1 (4.8)	–	0	–	3 (8.6)	–	0
OVERALL^b^	*N* = 1130		*N* = 598		*N* = 1133		*N* = 1120	
- Severe	331 (29.3)		165 (27.6)		324 (28.6)		327 (29.2)	
- Moderate	782 (69.2)		421 (70.4)		794 (70.1)		771 (68.8)	
- Mild	16 (1.4)		12 (2.0)		15 (1.3)		22 (2.0)	
- None	1 (0.1)		0		0		0	

^a^n(%) shown

^b^Intention-to-treat population

### Rescue and recurrence timing

**Fig. 1:**
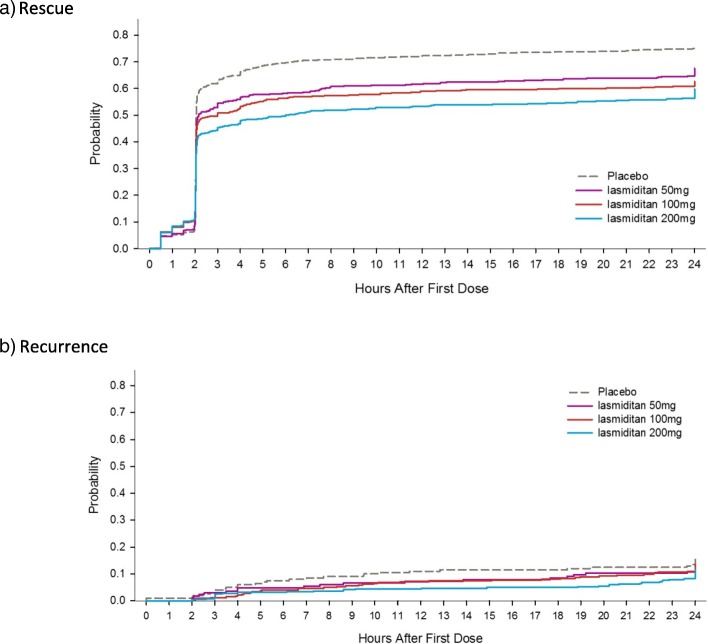
Time from first dose to second dose of study drug for **a** patients who were not pain free at 2 h and took a second dose of study drug or other migraine medication for rescue and **b** patients who were pain free at 2 h and took a second dose of study drug or other migraine medication for recurrence

In patients who did not achieve pain freedom at 2 h and who took a second dose for rescue, the median time to second dose was 2 h from first dose for all dose groups (per protocol, 2 h was the earliest time point at which a second dose of study medication was permitted.) In patients taking a second dose for recurrence, the median time to second dose for recurrence ranged from 7.0 h, for placebo, to 19.3 h, for lasmiditan 200 mg. (Table 4, Fig. 1).

**Table 4:** Time (in hours post first dose) of rescue and recurrence treatment, second dose intention-to-treat population

	Placebo	Lasmiditan 50 mg	Lasmiditan 100 mg	Lasmiditan 200 mg
RESCUE, n	606	245	388	310
- 25th percentile	2.0	2.0	2.0	2.0
- Median	2.0	2.0	2.0	2.0
- 75th percentile	2.0	2.0	2.0	2.6
RECURRENCE, n	21	13	35	28
- 25th percentile	4.0	7.6	4.5	8.0
- Median	7.0	11.3	9.5	19.3
- 75th percentile	11.5	18.4	18.8	22.1

*Abbreviation*: *n* number of patients

### Pain recurrence as defined in triptan studies

To enable comparison of the pain recurrence findings with lasmiditan to those of triptans, we performed post hoc analysis using data from SAMURAI and SPARTAN with recurrence defined using criteria previously employed in triptan studies (i.e., patient achieved none or mild pain at 2 h after first dose and subsequently reported moderate or severe pain [4]), 14–15% experienced pain recurrence with a lasmiditan dose versus 17% with placebo (Table 5).

**Table 5:** Pain recurrence after first dose as defined in triptan studies^a^

FIRST DOSE →	Placebo	Lasmiditan 50 mg	Lasmiditan 100 mg	Lasmiditan 200 mg
None or mild pain at 2 h, n	505	346	698	683
Recurrence of moderate or severe pain, n (%)	88 (17.4)	53 (15.3)	107 (15.3)	96 (14.1)

^a^Recurrence population defined as patients who achieved none or mild pain at 2 h after first dose and subsequently were recorded to have moderate or severe pain [4]

### Efficacy of second dose

**Fig. 2:**
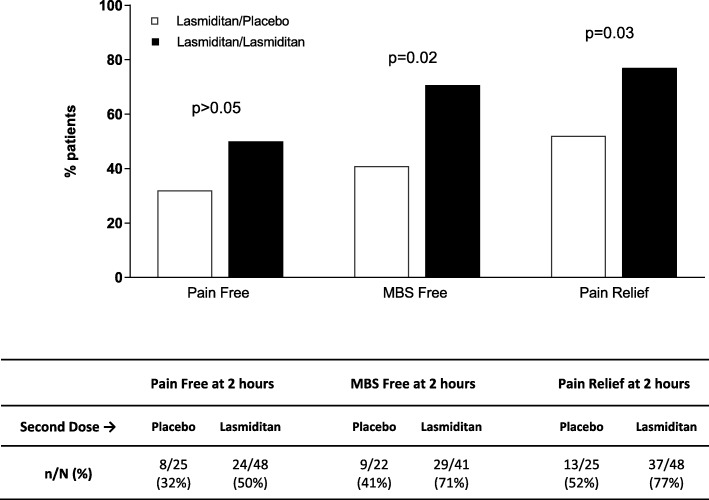
Outcomes at 2 h after second dose of study drug for the recurrence population

In patients taking lasmiditan as first dose, outcomes (pain free, MBS free, pain relief) at 2 h after a second dose for rescue was similar whether the second dose was lasmiditan or placebo for all lasmiditan dose groups (*p* > 0.05 in all cases) (Table 6). In patients taking lasmiditan as first dose, a greater proportion of patients taking lasmiditan versus placebo as second dose for recurrence were MBS free or achieved pain relief outcomes at 2 h (Fig. 2).

**Table 6:** Outcomes at 2 h after second dose of study drug for the rescue population

FIRST DOSE	Lasmiditan 50 mg		Lasmiditan 100 mg		Lasmiditan 200 mg	
SECOND DOSE	Placebo	Lasmiditan 50 mg	Placebo	Lasmiditan 100 mg	Placebo	Lasmiditan 200 mg
Pain free, n/N (%)	18/79 (23%)	33/166 (20%)	31/125 (25%)	67/262 (26%)	27/103 (26%)	59/207 (29%)
MBS free, n/N (%)	23/69 (33%)	52/142 (37%)	36/109 (33%)	74/221 (34%)	34/90 (38%)	65/182 (36%)
Pain relief, n/N (%)	36/79 (46%)	75/166 (45%)	60/125 (48%)	121/262 (46%)	49/103 (48%)	101/207 (49%)

*Abbreviation*: *MBS* most bothersome symptoms

### Safety findings

The proportion of patients with at least one TEAE after the second dose was similar whether randomized to placebo or lasmiditan as the second dose (Table 7). The most frequently reported TEAEs after second dose were dizziness, paresthesia, fatigue, asthenia, nausea and somnolence.

**Table 7:** Treatment emergent adverse events reported after second dose

Second Dose →	Placebo	Lasmiditan 50 mg		Lasmiditan 100 mg		Lasmiditan 200 mg	
	Placebo	Placebo	Lasmiditan 50 mg	Placebo	Lasmiditan 100 mg	Placebo	Lasmiditan 200 mg
Rescue population^a^	60/669 (9.0)	12/79 (15.2)	31/166 (18.7)	25/142 (17.6)	55/299 (18.4)	27/119 (22.7)	45/235 (19.1)
Recurrence population^a^	2/29 (6.9)	0/4	2/9 (22.2)	1/10 (10.0)	3/36 (8.3)	3/16 (18.8)	3/27 (11.1)

^a^Patients with ≥1 treatment-emergent adverse event after second dose, n/N (%)

## Discussion

We present the second dose efficacy and safety findings from 2 phase 3 placebo-controlled studies of lasmiditan for acute treatment of migraine, in which patients could take a second dose of study drug for rescue or recurrence. Fewer patients taking lasmiditan (at any dosage) versus placebo as first dose took a second dose of study medication, and the difference between active drug and placebo increased with increasing lasmiditan dose.

The proportion of a study population taking rescue medication at 2 h can be a useful secondary efficacy measure, reflecting the patient’s judgement of the efficacy of the investigational drug [9]. In the present study, of those patients taking lasmiditan as first dose (any dose), 68% had mild, moderate or severe pain at 2 h (versus 82% for placebo as first dose); of those patients with pain at 2 h, 48% took a second dose for rescue (versus 66% for placebo as first dose).

Acute treatments that are associated with lower recurrence rates have been shown to be more cost-effective, primarily because the total cost per treated attack is highly correlated with the need for a second dose [1]. In the current study, of those patients taking lasmiditan as first dose, overall 32% were pain free at 2 h; this is similar to what has been previously reported with use of oral triptans [3, 4] Of those who were pain free at 2 h, 7–10% took a second dose for recurrence of symptoms. Direct comparison of these findings with those of other acute treatments is somewhat problematic due to differences in the definitions across studies. Prior studies have considered the pain recurrence population to be either patients who were *pain free* or those who *achieved pain relief* (from moderate or severe pain to none or mild pain) at 2 h after first dose and subsequently experienced *moderate or severe pain*; in the current lasmiditan studies, the criteria for treatment for recurrence were pain free at 2 h and then recurrence of any pain (mild, moderate or severe).

In order to provide some level of comparison with other acute migraine therapies, we performed a sensitivity analysis using an alternate definition of recurrence to be consistent with prior studies [4]. In SAMURAI and SPARTAN 14–15% experienced pain recurrence with lasmiditan, across doses. In a meta-analysis by Ferrari et al. [4], some 30% of individuals who achieved pain relief at 2 h reported moderate or severe headache pain at 2–24 h, with some differences between individual triptans. Recurrence of headache pain was lower with eletriptan 40 mg or 80 mg (~ 20%). In a study of the effectiveness of eletriptan in Excedrin nonresponders, 9% of those who responded at 2 h took a second dose for recurrence of moderate or severe pain [10]. In SAMURAI and SPARTAN, 9% took a second dose for recurrence of moderate or severe pain.

We assessed the effectiveness of a second dose of lasmiditan. There was no evidence that a second dose of lasmiditan was effective for treating migraine in patients who were *not* pain free at 2 h (i.e., rescue treatment); since the second dose of study medication was either the same dose of lasmiditan as the first dose or placebo, we do not know if patients would have responded to a higher dose of lasmiditan for rescue. Redosing with sumatriptan or eletriptan has not been shown to be effective for patients who failed to respond to the first dose [11]. In patients who took a second dose for pain recurrence, there was some evidence that a second dose of lasmiditan may offer benefit over placebo for freedom from MBS and pain relief at 2 h; the percentage of patients who were pain free at 2 h was numerically greater with lasmiditan versus placebo as second dose, but the difference did not reach statistical significance. Triptans have also been shown to demonstrate efficacy of second dose to treat recurrence of symptoms [12–14]. Using the same definition of recurrence for the lasmiditan studies as for prior triptan studies, we found the effectiveness of treatment for pain recurrence was similar to that with triptans (data not shown).

There was no evidence of a worsened safety profile with a second dose; the frequency of TEAEs with a second dose of lasmiditan was similar to that for placebo as second dose. The most frequently reported TEAEs after the second dose were consistent with those most frequently reported TEAEs after the first dose [6, 7].

There are limitations to this study. The analyses were exploratory in nature, and no adjustments were made for multiple comparisons. Few patients took a second dose for recurrence (97 in total), resulting in limited statistical power to detect treatment group differences; data from individual lasmiditan dosing groups were pooled to address this. Patients were randomized to their second dose at the screening visit rather than being independently randomized at the time of taking a second dose for rescue or recurrence. Independent randomization would be difficult to implement since patients took the study medication as outpatients.

## Conclusion

In conclusion, there was no clear benefit of a second dose of lasmiditan for rescue treatment. A second dose of lasmiditan showed some evidence of efficacy when taken for headache recurrence. The incidences of TEAEs were similar whether the second dose was lasmiditan or placebo.

## Data Availability

Data are available to request 6 months after the indication studied has been approved in the US and EU. For details on submitting a request, please see the instructions provided at www.clinicalstudydatarequest.com

## Abbreviations

AE: Adverse event
BMI: Body mass index
CI: Confidence interval
ITT: Intention-to-treat
MBS: Most bothersome symptom
OR: Odds ratio
PGIC: Patient Global Impression of Change
TEAE: Treatment-emergent adverse event
